# Real-time Image Processing for Microscopy-based Label-free Imaging Flow Cytometry in a Microfluidic Chip

**DOI:** 10.1038/s41598-017-11534-0

**Published:** 2017-09-14

**Authors:** Young Jin Heo, Donghyeon Lee, Junsu Kang, Keondo Lee, Wan Kyun Chung

**Affiliations:** Pohang University of Science and Technology (POSTECH), Mechanical engineering, Pohang, 790-784 South Korea

## Abstract

Imaging flow cytometry (IFC) is an emerging technology that acquires single-cell images at high-throughput for analysis of a cell population. Rich information that comes from high sensitivity and spatial resolution of a single-cell microscopic image is beneficial for single-cell analysis in various biological applications. In this paper, we present a fast image-processing pipeline (R-MOD: Real-time Moving Object Detector) based on deep learning for high-throughput microscopy-based label-free IFC in a microfluidic chip. The R-MOD pipeline acquires all single-cell images of cells in flow, and identifies the acquired images as a real-time process with minimum hardware that consists of a microscope and a high-speed camera. Experiments show that R-MOD has the fast and reliable accuracy (500 fps and 93.3% mAP), and is expected to be used as a powerful tool for biomedical and clinical applications.

## Introduction

Flow cytometry (FC) allows high-throughput cellular analysis at single-cell resolution. The method has enabled analysis of large cell populations, and has been successfully applied in a variety of applications such as diagnostic medicine, immunology, and related areas^1^. Its extension, imaging flow cytometry (IFC), combines conventional FC with microscopy to enable analysis of heterogeneous cell population at both high throughput and high spatial resolution^2^. IFC acquires high-resolution single-cell images while conducting multiparametric analysis of high-volume cell populations, and the acquired rich information is used in various biomedical applications such as detection of live cells among dead cells and apoptotic bodies^3^, of adherent platelets/platelet fragments^4^, and of circulating tumor cells^5, 6^, An IFC platform with a miniaturized and disposable microfluidic device enables parallelization for high-throughput and high-volume analysis, and broadens applications of FC^2, 7^.

There are many studies that have considered IFC systems; for convenience we can divide them into two categories: development of 1) single-cell imaging systems capable of acquiring single-cell images at high speed, and of 2) image-processing methods capable of analyzing the acquired microscopic single-cell images. For single-cell imaging systems, many studies have tried to achieve both high throughput and high spatial resolution of imaging-in-flow systems^6, 8, 9^. Because acquisition of a high-quality image of a flowing cell is extremely difficult due to motion blur, the studies achieved high-throughput IFC by adding a specialized light source and additional detectors to conventional FC. For example, the commercialized IFC ‘ImageStream’ uses charge-coupled device (CCD) cameras and applies a time-delay integration (TDI) technique^6^. To achieve accurate cell focusing and tracking, ImageStream uses precise pumps, an in-line air chamber, and a velocity-detection subsystem for closed-loop control of the TDI readout rate. These additional subsystems increase the system’s versatility, but greatly increase its complexity and cost.

Many studies of image-processing methods to analyze single-cell images have applied image segmentation or machine-learning algorithms to reveal cell phenotypes or to quantify cellular DNA content^10–13^. One study used supervised learning to perform label-free quantification of DNA content and identification of phases in the cell cycle^11^; the authors used ImageStream to collect bright-field and dark-field images of cells in flow, then used commercial software packages and tools such as CellProfiler to analyze cell images^12^. Another study focused on integrating imaging technology with deep-learning technology to realize IFC^13^; a time-stretch quantitative phase-imaging system obtained quantitative phase and intensity images in real time, and used integrated feature extraction and deep learning algorithm to achieve label-free classification of cells and to detect cancerous cells. However, these studies acquired or analyzed cell images off-line as a post-experiment process; the number of images that can be stored for such analysis is limited by memory capacity. The ability to acquire, store, and analyze large numbers of cell images in real time is the biggest challenge in development of IFC systems that can analyze large-volume samples at high throughput^2^.

**Figure 1:**
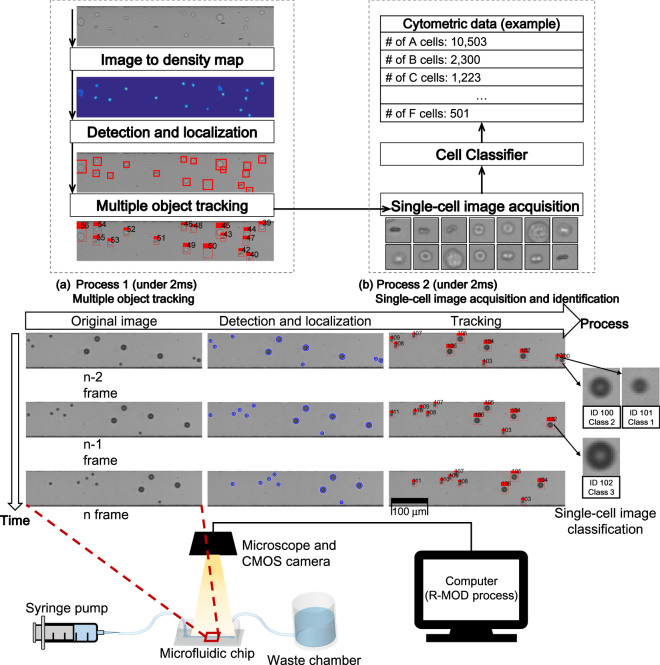
Schematic of the designed system and R-MOD. CMOS camera on microscope observes the microfluidic channel through which cell suspension flows. Bright-field microscopic image taken by the CMOS camera is represented by a grayscale image. The resolution of the image is 100 by 500 pixels and the CMOS camera collects image sequences at 500 frame/s (i.e. every 2 ms). Thus, R-MOD must perform the whole process in <2 ms. Two processes are executed in parallel (multi-threads) and each process should perform its task <2 ms. (**a**) Process 1 performs detection/localization and multiple object tracking. For the detection and localization tasks, FCRN converts the original grayscale images to probability density maps (<1.5 ms), then the Flattening algorithm extracts centre positions and sizes from the maps (<0.1 ms). After obtaining location of each cell, the multiple-object tracking algorithm finds correspondences between consecutive frames (<0.2 ms); this step eliminates repeated counting of cells in flow. (**b**) Process 2 performs single-cell image acquisition and identification. Using the tracking result, single-cell images can be obtained by cropping them from the original ROI image frame without duplications. The cropped single-cell images are evaluated by an image classifier based on a supervised learning to identify cell type.

Here we present a real-time image processing pipeline called R-MOD (Real-time Moving Object Detector) to acquire and analyze images of single-cells in flow for miniaturized microscopy-based label-free IFC (Fig. 1). The designed system can acquire single-cell images and analyze them in real time by applying image processing and machine learning to image sequences obtained by a microscope and a CMOS camera. R-MOD uses a multiple-object-tracking algorithm to count cells and to obtain an image of each single cell, then a supervised machine-learning algorithm analyzes the single-cell images to achieve label-free classification. Both single-cell image acquisition and analysis can be performed in real time (<2 ms) thanks to the pipeline’s low computational cost, so huge amount of acquired images need not be saved in memory for post processing; therefore, high-throughput and high-volume sample analysis is possible. Because counting and identification are both accomplished using deep-learning technology, which uses a convolutional neural network (CNN)^14^, R-MOD can detect and classify multiple cells and is insensitive to light and focus conditions of the microscope. As multiple cells can be detected simultaneously, the system can maintain high throughput at low flow rate by increasing concentration of cells. Thus, the proposed system can realize features of real-time IFC without additional subsystems such as precise pumps and velocity detector, which are required to generate a cell stream and to capture the images of cells under high flow rate. We conducted experiments using a suspension of mixed-size micro-particles and a sample of real biological cells to evaluate and validate the proposed system.

## System Configuration and R-MOD Pipeline

**Figure 2:**
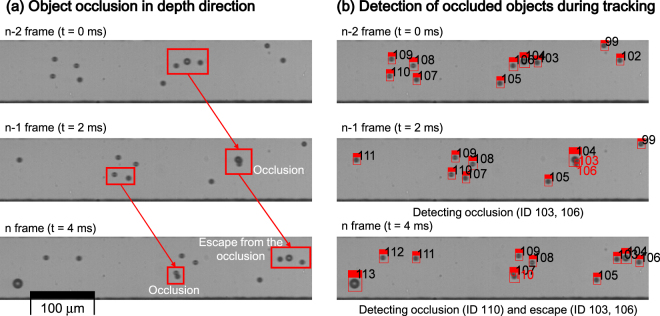
Occlusion mechanism and detection of the occlusion by tracking algorithm. Occlusion occurs due to overlap of several objects in the depth direction. If two objects are located at different depth positions, velocities of the two objects are different because Poiseuille flow has a parabolic velocity profile that is fastest at the centre and slowest at the wall. Thus, objects are occluded for only short times, then quickly escape the occlusion. (**a**) occurrence of occlusion at 2 ms. (**b**) the proposed tracking algorithm detected occluded objects (red circles) and reassigned objects that had escaped the occlusion (ID 103 and 106).

The experiment was conducted using a polydimethylsiloxane (PDMS) microfluidic chip that includes a straight micro-channel with 50 µm height and 100-µm width. The microfluidic chip has one inlet and one outlet; a syringe pump injects a cell suspension at constant flow rate into the chip. A microscope observes a region-of-interest (ROI) of size 100 × 500 pixels at downstream of the channel, and a camera acquires images of cells that pass through the ROI. The system uses a basic bright-field microscope and a CMOS-based high-speed camera to obtain images of cells flowing in the micro-channel; the obtained images are sent to a computer for the R-MOD process in real time. R-MOD is mainly composed of two parts: (**1**) multiple object tracking (Fig. 1a) and (**2**) single-cell image acquisition and identification (Fig. 1b). The two parts are processed in parallel using multiple threads and each part completes a given task within 2 ms to achieve 500 fps process speed.

## Multiple-Object Tracking

Main purpose of multiple-object tracking is precise cell counting by preventing re-counting of cells in flow that appear several times in the image sequence (depending on the flow rate and frame rate); the tracking result yields one single-cell image per cell without duplication. Multiple-object tracking entails (i) image segmentation, (ii) detection and localization, and (iii) tracking.

(i) *Image segmentation* simplifies the original image to reduce the complexity of image processing. Light and focus conditions vary among microscopy experiments, and in addition to the cells that are to be detected, the images contain noise and debris. These contents can degrade the precision of naïve image-processing algorithms such as blob detection, but a well-trained deep neural network can precisely simplify the original images into segmented image, regardless of experimental conditions and image noise (Figs S1 and S3). As a segmentation method to detect objects reliably, we used a convolutional neural network. The segmentation performs a real-valued regression that converts a grayscale microscopy image to a probability-density map, and thereby makes the detection and localization tasks easy and insensitive to noise (Fig. 1a ‘Image to density map’). Here, the probability density map **Y** is represented as a two dimensional mixture of Gaussians without a mixing coefficient:
  1 $${\bf{Y}}=\sum _{k=1}^{K}N((i,j)|{\mu }_{k},{{\rm{\Sigma }}}_{k}),$$
  where (*i*, *j*) is pixel index of the input grayscale image matrix, and *K* is the number of Gaussians in the density map. This segmentation procedure represents each cell in the original image as a bivariate $N((i,j)|{\mu }_{k},{{\rm{\Sigma }}}_{k})$ represents bivariate Gaussian distribution with mean **μ** and isotropic covariance (Σ = σ**I**). The means refer to centre position of each cell and the standard deviations refer to a third of the radius of each cell (*i.e*., r = 3σ); i.e., in a density map, each Gaussian distribution represents each cell in the original grayscale image as mean (position) and variance (size). To perform this conversion, we used a fully-convolutional regression network (FCRN) which was originally developed to count cells on petri dishes^15^; we modified the original FCRN structure to be appropriate for our fast microfluidic-microscope IFC system (Table S1).
  After a microscopic image is converted to a density map, its means and variances are extracted for detection and localization.
(ii) *Detection and localization* is the process of finding an arbitrary number of objects in an image (Here, the ‘detection’), and the determining the exact locations of the objects in the image (‘localization’) (Fig. 1a ‘Detection and localization’). Because segmentation has converted the original image to a probability-density map, detection and localization tasks become finding the means and variances in the probability density map; this task is much easier than detecting and localizing objects in the original microscopic image. We therefore developed an algorithm called ‘Flattening’ that can quickly extract means and variances from a probability density map; i.e., the input of flattening is a probability density map outputted by the FCRN regression, and the output is the set of means and variances (positions and sizes of arbitrary number of cells) in the map. Flattening first finds the maximum pixel value and its index in the given density map matrix **Y**; this index is a mean **μ** of the Gaussian distribution that has the smallest variance. The pixel value itself is the density *p*_max_ of the Gaussian distribution, and its variance σ^2^ can be obtained as follows (derivation in Supplementary Information S2):
  2 $${\sigma }^{2}=0.5\pi {p}_{\text{max}},$$
  This process obtains **μ** and σ^2^ of one Gaussian, then the obtained **μ** and σ^2^ is used to guide removal of this Gaussian from the density map by converting its pixel values to zeroes; this process is called flattening. The flattening process is repeated until all Gaussian distributions in the probability density map have been removed. This process yields all means and variances in the map. Detection and localization of an arbitrary number of objects in an image is a difficult problem, but Flattening can solve simply and rapidly (Pseudo-code of the flattening algorithm is shown in Algorithm S1).
  After the detection and localization tasks are completed, the tracking algorithm is executed (tracking-by-detection framework).
(iii) *Tracking* begins by finding corresponding detected objects in consecutive images to follow the motions of the moving objects (Fig. 1a ‘Multiple object tracking’). The tracking algorithm in the proposed pipeline is designed to detect occlusion quickly by considering the characteristics of the fluid flowing through the micro-channel.

The tracking algorithm assigns detected objects to a *track* variable that represents the object during an image sequence. The correspondence between detected objects in consecutive frames is found by solving an assignment problem based on the motions of flowing objects. An assignment problem is to find an optimal weight matching in a bipartite graph; we utilized a standard Hungarian algorithm to associate the detected objects and tracks. If a detected object matches one of the tracks, the detection is assigned to the track and the track continues until the track exits the ROI (assigned case). Four unassigned cases also occur; *in* (cell newly enters the ROI), *out* (cell leaves the ROI), *occlusion* (cell is hidden by another cell), and *appear* (hidden cell appears by escaping the occlusion). These four *states* correspond to case in which track and detection do not match one-to-one. If a detected object cannot be assigned to a corresponding track, the object is a new one that has entered the ROI (*in*) or has escaped from an occlusion (*appear*). If a track cannot find a corresponding detection, the track has exited the ROI (*out*) or has disappeared from it (*occlusion*). These states can be distinguished based on the motion of Poiseuille flow: each cell has constant velocity to the axial direction and barely moves to lateral direction. When occlusion occurs, most object detection algorithms, including Flattening, fail to detect objects. However, the state information (one assigned case; four unassigned cases) of our tracking algorithm gives us information about existence and absence of object occlusion; this information is used in next single-cell image acquisition step to filter out occluded objects. To achieve this occlusion detection, each track variable should have its associated state history during each time step. Poiseuille channel flow has a parabolic velocity profile, so the velocities of two objects are different if they are at different positions in the depth direction (*occlusion* state). Because of these physical laws, the overlap of two objects in an image sequence occurs only at a specific moment; this phenomenon is also evident in the experimental results (Fig. 2a). Thus, the *occlusion* state does not continue for as long as the time taken by the cell to pass through the ROI, and our tracking algorithm can successfully track multiple objects and detect occlusion (Fig. 2b). State history of a track variable is used to find a clear image that does not include an occluded object.

To count and distinguish all objects, every track has its unique ID, which is assigned when a new track is created; i.e., when a new cell enters the ROI. Cell ID numbers are assigned sequentially from 1, so the last ID is the number of cells detected. By using the track ID, the tracking algorithm prevents recounting of cells that have been captured several times in the image sequence; this process makes the pipeline can acquire one single-cell image for each cell. Finally, each track variable contains ID, sequential positions, and state history of the object; these data will be used in the next cell-identification step; therefore, when a cell leaves the ROI, the cell’s corresponding track variable is stored temporarily in a fixed-size circular buffer.

## Single-Cell Image Acquisition and Identification

This process acquires a single-cell image by cropping a patch of each detected cell with a certain size in the ROI, then applies a cell-classification algorithm to identify the cell-type of the single-cell image (Fig. 1b). When a cell leaves the ROI (*out* state occur), the process searches the state history of the leaving cell’s track to find the image frame in which the leaving cell is most clearly captured (without occlusion), then crops a single-cell image based on the cell’s centre position. We fixed size of cropped single-cell images as 21 × 21 pixels. This cropping process to obtain a clearly-captured single-cell image requires two fixed-size circular buffers that store a certain number of past ROI images and tracks. The cropped single-cell images are also stored in another buffer to send it to the classifier; the identification step applies a cell-classification algorithm to these images to identify cell types in real time. Because R-MOD applies the classification algorithm and identifies cells in real time, the images need not be stored for post processing, so high-throughput online single-cell analysis is possible. As the classifier, we used an image classifier based on convolutional neural network (CNN) which can perform automatic feature extraction^14^. Many classifiers based on CNNs can identify cell types from microscopic cell images^15–17^ and R-MOD can use any image classifier to identify the acquired single-cell image at the end of the pipeline. As the main contribution of this study is to obtain single-cell images and to identify them in real time for high-throughput IFC using only an imaging process without additional hardware, we used a simple CNN classifier on this study to show the feasibility of R-MOD as an IFC platform.

## Results

To validate the proposed R-MOD pipeline, we performed experiments using two different sample solutions: 1) mixed-size micro-particles for accurate control of sample concentration to quantitatively evaluate accuracy, and 2) real cells to validate its feasibility for practical biological applications. Several experimental conditions including flow rate and cell concentration should be considered before performing the experiments. Considering the computational speed of R-MOD, the frame rate of the camera is fixed at 500 frames/s, and shutter speed is set to 1/10000 s. Flow rate has a trade-off relationship with image quality. High flow rate (fast cell velocity) can raise throughput but induce motion-blur that can degrade the quality of images, whereas low flow rate (slow cell velocity) yields clear images but reduces throughput. This trade-off occurs when the cell flow forms only a single stream. However, since R-MOD can trace multiple cell flow regardless of their lateral positions, we do not need to generate a single stream. Therefore, we could increase the concentration of cell suspension to generate randomly distributed cell flow and decrease the flow rate to obtain high-quality image of cells while preserving throughput; i.e., Throughput [count/s] ~ Flow rate [ml/s] × concentration [count/ml].

In the micro-particle experiments (Supplementary Information Multimedia), we used polystyrene micro-particles with different diameters of 7, 10, or 15 μm, each at 0.3% w/w in a mixed suspension. Theoretically, the number concentration of the mixed suspension is 7,700 beads/μL and number ratio of each bead is 100/34/10 (7/10/15-μm). A syringe pump injected the suspension with constant flow rate. If flow rate is 10 µL/min, then throughput is estimated as 1,283 beads/s.

Using these experimental conditions, three experiments were performed and each experiment recorded 2,000 consecutive images during the experiment for 4 s at 500 fps (Table 1). For the Exp-1, flow rate was 6 μL/min and estimated throughput is 770 cells/s. R-MOD detected 1,540 beads with 7-μm diameter, 549 beads with 10-μm diameter, and 257 with 15-μm. The number ratio was 100/36/17, which is similar to the theoretical number ratio of 100/34/10 calculated based on the concentrations of particles in the suspension. This statistical analysis is only a rough comparison. As a check of tracking accuracy, the counting result was compared with counts obtained by six human participants who viewed a recorded image sequence; it took about 30 minutes to 1 hour per person for 2,000 images. Counting result by R-MOD was 2,346; the mean manual counting result was 2,343 with a standard deviation of 6.3; i.e., the difference between R-MOD counts and human counts was 3 (relative error 0.128%). The estimated throughput was 770 cells/s, but actual throughput was ~600 cells/s because particles are not uniformly distributed in the microfluidic channel and connecting tube. To show more cases, Exp-2 and Exp-3 were also performed by changing flow rate (Table 1). Actual throughputs of Exp-2 (10 μL/min) and Exp-3 (15 μL/min) are 776 beads/s and 1,340 beads/s, respectively.

**Table 1:** Mixed size micro-particle experiments.

Experiments		Human counting	R-MOD counting	
	Duration	Count (mean ± s.d.)	Count (7/10/15-μm) Throughput	Number ratio (est. 100/34/10)
Exp-1 (6 μL/min)	4 sec	2,343 ± 6.3	2,346 (1,540/549/257) 587 cells/s	100/35/10
Exp-2 (10 μL/min)	4 sec	—	3,103 (2,423/462/218) 776 cells/s	100/19/9
Exp-3 (18 μL/min)	4 sec	—	5,362 (3,055/1,306/1001) 1,340 cells/s	100/43/33
Exp-Large (18 μL/min)	17 min	—	635,844 (396,122/133,891/105,831) 616 cells/s	100/34/27

From Exp-1 to Exp-3, 2,000 recorded images (during 4 sec) were processed by R-MOD. To quantitative evaluation of counting results, six human participants counted flowing beads by viewing the recorded image sequence of Exp-1. Error of counting result between human and R-MOD is 3. Since beads in the suspension are not uniformly distributed, every experiment shows different actual throughput (from 600–1,300 cells/s). Exp-Large analysed a large number of images (>510,000) during 17 minutes to show the high-throughput and real-time capability of the proposed system. Number ratio is calculated based on the number concentration of the mixed bead to roughly compare the actual detected counts.

**Figure 3:**
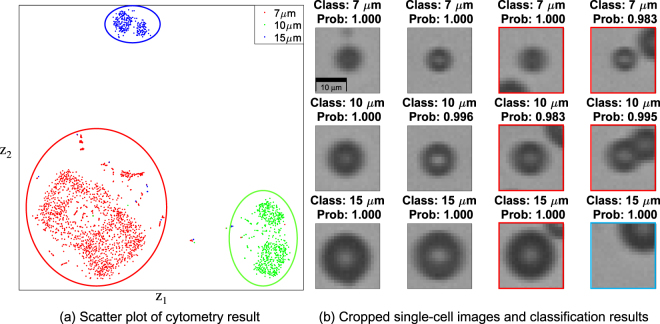
(**a**) Scatter plot of cytometry result of mixed-size bead suspension. t-SNE embedding was applied to reduce the high dimensional feature map to two dimensions for visualization. The output of the last hidden layer has originally 300 dimension, and t-SNE embedded its dimension to depict the classification result. Each axis represents the latent space (**z**) variable embedded by t-SNE. Point colors are labelled according to its classes: 7 μm (red): 1,540 points, 10 μm (green): 549 points, 15 μm (blue): 257 points. (**b**) Cropped single-bead images and their classification results. Some cropped single-cell images include another bead (doublet; red boxes) but the classifier predict category of the object near the centre position with high probability. This property can filter out doublet. In addition, cyan box shows a case that the object is not located at the centre position but classifier can predict its class due to the invariance of convolutional neural networks to small translation.

The cytometric result obtained from the Exp-1 was processed using t-distributed stochastic neighbour embedding (t-SNE)^18^ for dimensionality reduction to visualize the result as a scatter plot (Fig. 3a). Each point represents the classification result of one cropped single-cell image (Fig. 3b). The scatter plot shows that each class is well distinguished by the classifier, so its accuracy is sufficient to identify the mixed beads in this experiment. Some untargeted cells were included in the cropped single-cell image because two objects were close together (Fig. 3b red boxes). In this case (which is called a doublet), the classifier predicts the category of the cell located near the centre of the image with a high probability, and excludes the other cell; the excluded cell is located at the centre of a different cropped single-cell image. In addition, even though the cell is not in the centre of an image, the classifier can also predict its class category because the CNN classifier is invariant to small translation (Fig. 3b blue box)^19^. Therefore, doublets and shifted objects are automatically corrected by the CNN classifier due to its strong feature-extraction capability.

The three experiments that analyzed 2,000 images were for quantitative evaluation of the proposed pipeline. To show the high-throughput and real-time capability of the system, we analyzed large sample of the mixed particles for long time. The same suspension was used and R-MOD analyzed the sample for 17 minutes with more than 510,000 images. Since the experiment analyzed huge data in real time without storing image sequence in a memory, the result cannot be evaluated quantitatively. The result shows 396,122 beads with 7-μm, 133,891 beads with 10-μm, and 105,831 beads with 15-μm; actual throughput is 616 cells/s and the ratio is 100/34/27 (Table 1. Exp-Large). This result shows the large-quantity analysis capability of the proposed system.

In addition, we compared the speed and accuracy of our pipeline with existing famous methods quantitatively^20–22^. The main task of the proposed pipeline is multiple-object detection and tracking to classify flowing cells in real time in a microfluidic channel. To do this comparative evaluation, we implemented Faster R-CNN^20^ which shows high precision, and Fast YOLO^21^ which shows fast detection speed on our mixed micro-particle experiment dataset (Table 2). Faster R-CNN and Fast YOLO use boxes that are hypotheses for object locations; if the number of boxes is large, precision increases but speed decreases. The flattening algorithm that R-MOD uses for object detection does not use hypothesis boxes. In the comparison, R-MOD was the fastest and had the highest precision. However, R-MOD is designed for fast and accurate detection and tracking of flowing cells, but cannot be applied to natural images like the PASCAL VOC dataset. We argue that the proposed pipeline is suitable for flowing cell applications. We also evaluated the pixel accuracy of the flattening algorithm (Supplementary Information S4).

**Table 2:** Comparison of existing object detection methods with the proposed pipeline.

Dataset	PASCAL VOC 2007 test^22^ (Natural images)			Flowing micro-spheres (Microscopic image, 100 × 500 pixels)		
Method	*mAP*	FPS	# boxes	*mAP* (0.4 IoU)	FPS	# boxes
Faster R-CNN^20^	62.1%	17	300	69.3%	21	2,048
Fast YOLO^21^	52.7%	155	98	40.3%	175	98
R-MOD (ours)	—	—	—	**93.3%**	**500**	—

Mean average precision (mAP) with 0.4 intersection of union (IoU) represents precision of object detection, and frames per second (FPS) represents computational speed. Left half of the table shows comparison results by applying object detection methods to natural images (PASCAL VOC 2007 dataset). Right half result is obtained by applying detection methods to our micro-particle experiment. Our R-MOD was the fastest and most precise.

**Figure 4:**
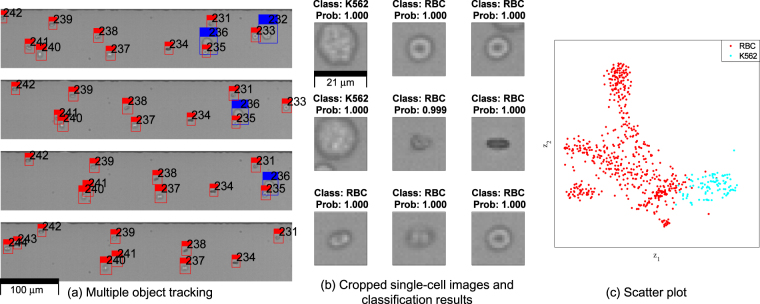
Experiment using K562 cells and human red blood cells (RBCs). (**a**) Multiple object tracking assigns IDs to each cell in flow (blue box: K562 cell; red box: RBC). (**b**) Each single-cell images acquired by R-MOD was evaluated by a classifier to identify the cell type (class and probability are shown above each image). Most identification show high prediction probabilities. (**c**) The scatter plot of the experiment. Two dimensional t-SNE embedded was applied to visualize the classification result. RBC (red) and K562 (cyan) show more distributed data than the mixed-particle experiment.

We also performed an experiment using live and fixed cells; live human red blood cells (RBCs) (Innovative Research Inc., USA) and fixed K562 cells (Femtofab, Korea) were mixed and appropriately diluted. The multiple object tracking successfully cropped single-cell images (Fig. 4a) and the classification results were highly accurate (Fig. 4b). RBCs are typically biconcave disks, so they show various shapes as they flow in the microchannel (round, ellipse, or rotated ellipse for live cells; distorted circle for dead cells). The training data of the classifier includes these differently-shaped RBCs, so the classifier can successfully identify K562 and RBCs with high precision. Except for the training data, the network structure and the learning method of the classifier were the same as in the bead experiment. The scatter plot of the cell experiment showed a wider distribution of data than obtained in the mixed-size particle experiment due to more complex morphologies (Fig. 4c). If the number of cell types to be identified increases or morphologies of cells are complex, the classifier should be replaced with a better model to improve the classification accuracy. The cell experiment shows that R-MOD can identify actual cell morphologies as well as spherical particles, so we expect it to have biomedical and clinical applications.

## Discussion

Imaging flow cytometry have enabled single-cell analysis that had both high throughput and high spatial resolution. IFC can measure rich information at single-cell resolution and identify complex cell phenotypes, but most previous research has focused on developing a novel optical system to achieve fast image acquisition^2^. In this study, we realized the single-cell image acquisition feature of imaging flow cytometry by developing a real-time image processing pipeline without complicated optical and mechanical subsystems. The R-MOD pipeline traces multiple cells in flow and acquires each cell image with high speed, and can therefore identify them in real time. In addition, this platform has a simple hardware configuration because the whole system is composed of only a common bright-field microscope, a CMOS camera, a microfluidic chip, and a desktop computer. The proposed system can determine the number and phenotype of cells in a heterogeneous population of large volume sample. Key points of our platform can be summarized as (1) capability for real-time analysis, (2) label-free analysis, (3) sheath-less and cell stream-free flow cytometry, and (4) parallel image analysis that enables both high throughput and high-quality image acquisition.

The R-MOD pipeline is related to automated microscopy^10^. Most previous studies related to image analysis of biological cells or tissue have applied image processing and machine-learning algorithms to images of cells on petri dishes, but our approach accurately counts and identifies ‘flowing cells’ in a live video stream of a large-volume sample, as in conventional flow cytometry. Automatic detection and tracking of multiple cells in flow are challenging tasks because most image-segmentation algorithms are very sensitive to parameters such as threshold value, and their precision degrades as the signal-to-noise ratio increases^23^. In addition, light condition and focal length can easily vary even under the same experimental setup, so conventional image segmentation processes entail tedious parameter adjustments. In contrast, our method shows consistent image processing results under various optical conditions, because of the consistent segmentation capability of deep convolutional neural networks. Moreover, R-MOD’s accuracies in detection, localization and tracking tasks are reliable enough to be used in many different microfluidic applications such as droplet sorting and multiple cell manipulation as well as IFC.

We also performed experiments to verify the speed and precision of the proposed platform. The mixed-size micro-particle experiment showed reliable speed (500 fps) and precision (93.3% mAP) of the proposed platform. The cytometric result obtained by R-MOD clearly represents each population of subgroup with different colours by the visualization method, so it does not require the manual gating process that is usually used in conventional flow cytometry. The live cell experiment showed the possibility of live/dead analysis as well as cell-type classification.

In the experiments, we used a simple CNN-based image classifier to identify cell types. The classifier accurately identified different size beads, and RBCs and K562 cells. The samples used are trivial and easy, so more-compelling classification tasks such as distinction of peripheral blood mononuclear cell from granulocyte seems to be required. However, the purpose of this paper is not to improve classification range and accuracy, and several studies have already performed image classification for various biological cells^15–17^. For example^17^, used a CNN classifier to identify types of white blood cells including basophil, eosinophil, lymphocyte, monocyte, and neutrophil based on low-resolution microscopic image. From this evidence, we can expect that a CNN classifier will be able to classify cells that have similar size but different morphology. Classification accuracy depends on several factors, including the dataset, the optimization method, and the structure of the neural network. Research into deep learning has provided various classification algorithms that can accurately infer a class category from an image, and their accuracy is increasing. The application range of R-MOD can be broadened and its identification accuracy can be improved by replacing the classifier at the end of the pipeline with a state-of-the-art classifier that has been trained on many relevant cell dataset. The resolution of acquired images also affects the accuracy of analysis, so adoption of a superior microscope and superior camera will also improve system’s analysis accuracy. We expect that R-MOD will have various applications such as label-free blood diagnosis and circulating tumor cell detection. In addition, R-MOD has great potential to be used for high-throughput IFC; we expect that the designed system will contribute to progress in IFC and in single-cell biology.

## Method

### Implementation of the system

The proposed platform consists of a CMOS-based high-speed vision system to acquire microscopic image at high speed, a computer to process the R-MOD pipeline, and microfluidic chip and syringe pump to generate constant flow rate. The imaging system is configured on an inverted microscope (IX73, Olympus Co.), and an objective has a numeric aperture (NA) of 0.3 and magnification of ×10. As a light source, halogen lamp is used with illumination filter for Köhler illumination. The high-speed vision system (Photron Co. Japan) consists of a high-speed CMOS camera and an FPGA board to send image sequences to the computer in real time. The microfluidic chip was fabricated using soft lithography^24^.

To implement R-MOD, we wrote custom code based on open-source library and toolbox. For the neural network implementation including FCRN and CNN, we used MatConvNet (http://www.vlfeat.org/matconvnet/) and Caffe (http://caffe.berkeleyvision.org/). We used MatConvNet to train the FCRN and CNN classifiers off-line. We used Caffe for feedforward computation during the experiment. Here, Caffe provides a GPU computation setting to accelerate the computing, and we used NVIDIA GTX 1080. To implement the flattening and tracking algorithms we wrote custom source code based on MATLAB and C++ language.

### Training dataset

Both FCRN and CNN classifier were trained using a pre-acquired dataset. The training dataset for FCRN is a set of original microscopic images (input) and its probability density map (label). To obtain label data of the FCRN training dataset, we manually pointed out the centre position and size of each cell in the microscopic image. Based on the centre position and size information, equation (1) can generate the probability density map. The training dataset for the CNN classifier is a set of single-cell images (input), each with its class number (label). Each single-cell image used in training data was labelled manually because micro-particles of different sizes, and K562 and RBC are sufficiently distinguishable by eye; however, for samples that are difficult to distinguish by eye, high-purity cell samples should be acquired to produce training data for a classifier.

### CNN Classifier

As a classifier, we used a convolutional neural network with a fully connected layer (Table S.2). The CNN classifier can automatically extract and recognize image features by training the network on an image dataset. The classification accuracy of the classifier depends on several factors, including the classification algorithm, training dataset, and optimization method. The CNN classifier that we used identifies the class of a 21-by-21 pixel single-cell image in <1 ms, and the size of single-cell images can be modified depending on the cell size. For the mixed micro-particle experiment, the micro-particles have simple features, so the simple CNN structure is used. If single-cell images that have many classes and features should be considered, fancy network structures should be employed for increasing the learning capacity.

## Electronic supplementary material

Supplementary Video

Supplementary Information
