# Supplementary material for: Real-time Image Processing for Microscopy-based Label-free Imaging Flow Cytometry in a Microfluidic Chip
Source: Sci Rep. 2017 Sep 14;7:11651. doi: 10.1038/s41598-017-11534-0 (PMC5599535; doi:10.1038/s41598-017-11534-0)
Supplement: Supplementary file 2 — Supplementary Information [file 41598_2017_11534_MOESM2_ESM.pdf]

# Real-time Imaging Processing for Microscopy-based Label-free Imaging Flow Cytometry in a Microfluidic Chip

Young Jin Heo[1], Donghyeon Lee[1], Junsu Kang[1], Keondo Lee[1] and Wan Kyun Chung[2]

## S.1. Fully convolutional regression network for image segmentation

The first part of CellNet is a real-valued regression from a grayscale microscopic image to a probability density map to make the detection and localization task easier (Fig. S.1). Here, a probability density map is represented as mixture of Gaussians without mixing coefficient:

$$\mathbf{Y} = \sum_{k=1}^{K} \mathcal{N}((i,j)|\boldsymbol{\mu}_k, \boldsymbol{\Sigma}_k) \tag{S.1}$$

where $(i, j)$ is pixel index of the input grayscale image matrix $\mathbf{X}$, $\mathbf{Y}$ is the output probability density map matrix, and $K$ is the number of Gaussians in the density map. $\mathcal{N}((i,j)|\boldsymbol{\mu}_k, \boldsymbol{\Sigma}_k)$ represents bivariate Gaussian distribution with mean ($\boldsymbol{\mu} = [\mu_x, \mu_y]^T$) and isotropic covariance ($\boldsymbol{\Sigma} = \sigma^2 \mathbf{I}_{2\times2}$). The mean vector refers to center position of each microsphere and the standard deviation refers to a third of radius of each microsphere (i.e., $r = 3\sigma$). We can estimate the number of microspheres in each image frame by summing its density map because each Gaussian distribution in the density map refers to a microsphere, and integral of a Gaussian distribution equals 1 [1]:

$$\sum_{i,j} Y_{i,j} = K. \tag{S.2}$$

Moreover, the simple representation (means and variances) of the density map enables easy detection of multiple objects using the *Flattening* algorithm that is discussed in Section S.2.

The fully convolutional regression network (FCRN) enables pixel-wise training and prediction, then the network produces same size of output as its input (i.e., $\mathbf{X} = \mathbb{R}^{H\times W}$ and $\mathbf{Y} = \mathbb{R}^{H\times W}$) [1], [2]. We modified the original FCRN and the network structure is shown in Table. S.2. Compared to the deep model from [1], the major distinction of our model is that isotropic Gaussian distribution is used as label data to obtain size information of objects. In the network structure, the convolution layers extract features in the input grayscale images, and the filter weights were randomly initialized without bias terms. The deconvolution layer performs upsamping to reconstruct size of output as the input size, and initial weights of the deconvolution filter were set to be bilinear interpolation filter without bias terms. The structure can be properly modified depending on the situation. As a loss function, we utilized Euclidean loss ($L_2$ distance or mean square error) as:

[1]The authors are with the Robotics Laboratory, school of Mechanical Engineering, Pohang University of Science and Technology (POSTECH), Pohang, 790-784, Gyung-buk, Korea {heoyoungjin,sansoveria}@postech.ac.kr

[2]Wan Kyun Chung is with Faculty of Mechanical Engineering, POSTECH, Hyojadong, The South Korea. wkchung@postech.ac.kr

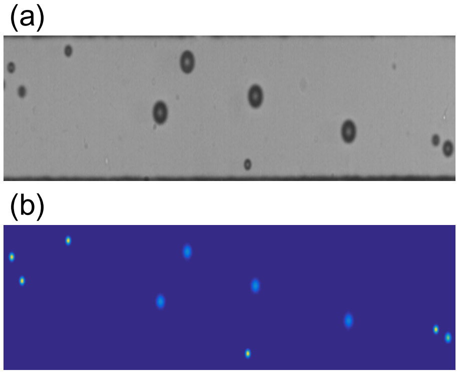


Fig. S.1. **Probability density map regression.** (a) Grayscale microscopic image (input of FCRN). Ten microspheres with different sizes are included in the image. (b) Probability density map (output of FCRN). Each microsphere is regressed by Gaussian distribution

$$L = \frac{1}{2N} \sum_{n=1}^{N} ||\mathbf{Y} - \mathbf{Y}_{label}||^2 \tag{S.3}$$

where $N = H \times W$. It should be noted that the input images have to be subtracted by the mean image.

To train the designed FCRN, stochastic gradient descent with weight decay was used as a back-propagation algorithm, and the weights were updated in stochastic mode. Learning rate of gradient descent was set to 0.001 during first 10 epochs, and then changed to 0.0001 in the remaining epochs. The weight decay was 0.0005. Label data was constructed by manually pointing out the center points and sizes of microspheres, and Gaussian distribution was computed based on these value. For the mixed micro-particle experiment, totally 200 images with different light conditions were used to train the network. During the training process, we monitored absolute error of density map integration (S.2), which is abs($K_{\text{real}} - K_{\text{predict}}$) to determine proper epoch of the optimization. As training epoch progressed, the integration error decreased, and predicted density map got similar to the label shown in Fig. S.1 (b) (Fig. S.2 (a)). Error did not show more convergence after the epoch 10 (Fig. S.2 (b)). Whole training process above was implemented using MATLAB and MatConvNet [3].

The FCRN enables robust detection of cells regardless

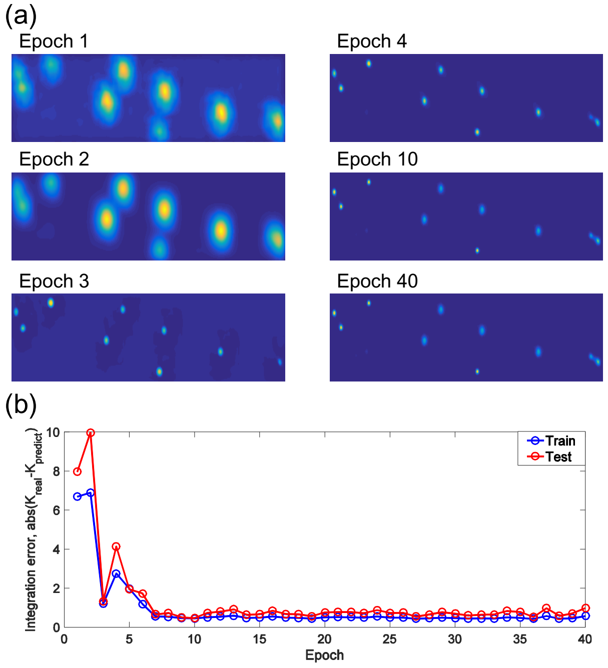


Fig. S.2. **Training and test result.** (a) As the number of epochs increase, trained output of FCRN resembles the label shown in Fig. S.1. (b) Integration error of both training and test process converge to under 0.3.

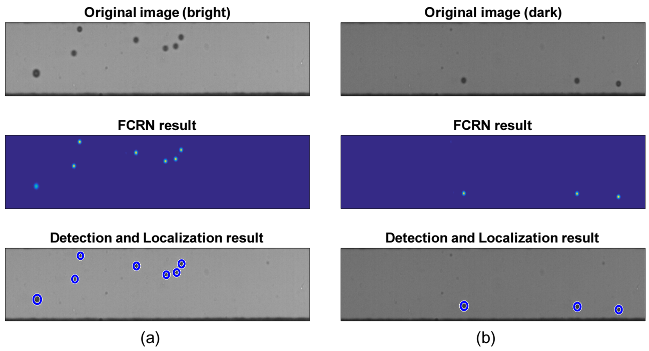


Fig. S.3. **Robustness of the FCRN regression.** FCRN regression and detect/localization results under the different light condition are compared to verify the robustness of the FCRN. Both bright (a) and dark (b) images are consistent.

of microscope light and focus condition by using training dataset obtained from various conditions. Naive image processing cannot consistently segment the image without changing the tuning parameters. Fig. S.3 shows the robustness of FCRN by comparing FCRN and Flattening results of different light conditions' original microscopic images.

## S.2. Flattening for Extraction of Means and Variances in Mixture of Gaussians

Flattening is a simple algorithm that can extract means and variances from a predicted density map. Complicated pattern recognition techniques are inappropriate for inference of means and variances because the number of objects changes in every image and is not exactly given. However, flattening algorithm which we have developed uses a fast and simple

TABLE S.1
Structure of FCRN

| No. | Layers | Size (filter, pool) | Stride | Pad | Input size ($H \times W \times D$) |
|---|---|---|---|---|---|
| 1 | Conv - ReLU | 3 | 1 | 1 | $100 \times 500 \times 1$ |
| 2 | Max pooling | 2 | 2 | 0 | $100 \times 500 \times 32$ |
| 3 | Conv - ReLU | 3 | 1 | 1 | $50 \times 250 \times 32$ |
| 4 | Conv - ReLU | 3 | 1 | 1 | $50 \times 250 \times 64$ |
| 5 | Deconv | 3 | - | - | $50 \times 250 \times 32$ |
| 6 | Conv - ReLU | 3 | 1 | 0 | $101 \times 501 \times 32$ |
| 7 | Conv - LRN | 2 | 1 | 1 | $99 \times 499 \times 16$ |
| 8 | Euclidean loss | - | - | - | $100 \times 500 \times 1$ |

* Note that ReLU is rectified linear unit, Conv is convolution, Deconv is deconvolution (or convolution transpose) and LRN is local response normalization.

**Algorithm S.1** Flattening algorithm

**Input:** $\mathbf{Y}$, probability density map
**Output:** $\boldsymbol{\mu}, \boldsymbol{\sigma}^2$, means and variances
1: $\tilde{\mathbf{Y}} = F(\mathbf{Y}, \epsilon)$
2: $[p_{\max}, \hat{\mu}] = \text{Max}(\tilde{\mathbf{Y}})$
3: **while** $p_{\max} \leq \epsilon$ **do**
4: $\quad \hat{\sigma}^2 = \frac{1}{2\pi p_{\max}}$
5: $\quad \boldsymbol{\mu} \leftarrow [\boldsymbol{\mu}; \hat{\mu}]$
6: $\quad \boldsymbol{\sigma}^2 \leftarrow [\boldsymbol{\sigma}^2; \hat{\sigma}^2]$
7: $\quad \tilde{\mathbf{Y}} \leftarrow \tilde{\mathbf{Y}} - \mathcal{N}(\hat{\mu}, \hat{\sigma}^2)$
8: $\quad [p_{\max}, \hat{\mu}] = \text{Max}(\tilde{\mathbf{Y}})$
9: **end while**

strategy for extracting means and variances of mixture of the arbitrary number of Gaussians (Algorithm. S.1).

The input is a probability density map predicted by the FCRN and the output is means and variances. In Algorithm. S.1, Line 1 performs thresholding to the predicted density map with $\epsilon = 0.1$ as

$$F(x, \epsilon) = \begin{cases} x & \text{if } x > \epsilon, \\ 0 & \text{otherwise} \end{cases}. \tag{S.4}$$

Here, the thresholding of the predicted map is an important step because empty space of predicted density is not exactly zero differently with label data (Fig. S.4). After performing the thresholding, main flattening loop is executed as explained in the manuscript. Here, the relationship of variance and maximum density is derived as follows:

$$\mathcal{N}(\mathbf{x}|\boldsymbol{\theta}) = \frac{1}{2\pi|\Sigma|^{1/2}} \exp\Big\{-\frac{1}{2}(\mathbf{x}-\boldsymbol{\mu})^T\Sigma^{-1}(\mathbf{x}-\boldsymbol{\mu})\Big\}, \tag{S.5}$$

$$\text{if} \quad \mathbf{x} = \boldsymbol{\mu} \quad \text{then} \quad \sigma^2 = \frac{1}{2\pi p_{\max}} \tag{S.6}$$

## S.3. Tracking Algorithm

Tracking part finds the correspondence of objects between two consecutive frames to trace multiple objects flowing in a micro-channel (Fig. S.5). To do this, we defined three vectors that represent positions and sizes of each object computed

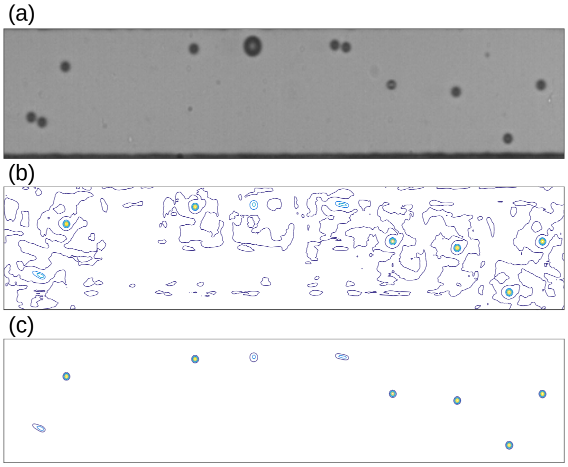


Fig. S.4. **Thresholding effect to the predicted density map.** (a) Original microscopic image. (b) Countour plot of the predicted density map. (c) After thresholding the predicted density map.

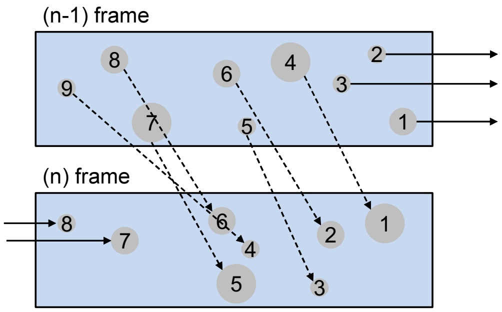


Fig. S.5. **Correspondences analysis of consecutive frames.** Several objects leave from the frame (1,2, and 3 in $n-1$ frame), several objects come into the frame (7 and 8 in $n$ frame), and remaining objects are matched to same objects based on the correspondence analysis.

from the flattening algorithm as follows:

$$\boldsymbol{p}_{x,n} = [\mu_{x,1}, \cdots, \mu_{x,K_n}]^T, \tag{S.7}$$
$$\boldsymbol{p}_{y,n} = [\mu_{y,1}, \cdots, \mu_{y,K_n}]^T, \tag{S.8}$$
$$\boldsymbol{s}_n = [\sigma_1^2, \cdots, \sigma_{K_n}^2]^T, \tag{S.9}$$

where $\boldsymbol{p}_{x,n}$ and $\boldsymbol{p}_{y,n}$ are $x$ and $y$ position vectors of $K_n$ objects at $n$ frame, respectively. $\boldsymbol{s}_n$ is the size vector of $K_n$ objects at $n$ frame.

It should be noted that Poiseuille flow has a parabolic velocity profile in which velocity is the fastest at the channel center and gets slower at the closer position to the channel wall. Therefore, lateral position of a particle is dominant to determine its velocity. We defined two principles based on the properties of Poiseuille flow: 1) there are minimum and maximum distance in the axial direction that objects can move within frame time interval, 2) there is small or no movement in lateral direction. In addition, size of a object does not change much, so the size vector can also be used to find correspondences. To formulate the principles, we first

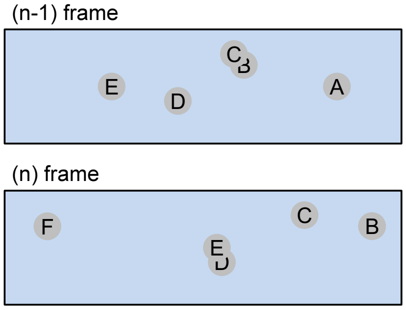


Fig. S.6. **'New' and 'vanishing' objects between consecutive frames.** Due to the occlusion, four states are defined: *in*, out, *appearance*, and *disappearance*.

define error matrices as follows:

$$\boldsymbol{D}_x := d(\boldsymbol{p}_{x,n}, \boldsymbol{p}_{x,n-1}) \in \mathbb{I}^{K_{n-1} \times K_n}, \tag{S.10}$$
$$\boldsymbol{D}_y := d(\boldsymbol{p}_{y,n}, \boldsymbol{p}_{y,n-1}) \in \mathbb{I}^{K_{n-1} \times K_n}, \tag{S.11}$$
$$\boldsymbol{D}_\sigma := d(\boldsymbol{s}_n, \boldsymbol{s}_{n-1}) \in \mathbb{R}^{K_{n-1} \times K_n}, \tag{S.12}$$

where $d(\cdot)$ refers to element-wise comparison matrix of two vectors using Euclidean distances as

$$d(\boldsymbol{a}, \boldsymbol{b}) = \begin{pmatrix} \text{abs}(a_1 - b_1) & \cdots & \text{abs}(a_N - b_1) \\ \vdots & \ddots & \vdots \\ \text{abs}(a_1 - b_M) & \cdots & \text{abs}(a_N - b_M) \end{pmatrix}. \tag{S.13}$$

Then, the principles are formulated as boolean matrices as follows:

$$\boldsymbol{B}_x := \big(\epsilon_{x,\text{min}} < \boldsymbol{D}_x < \epsilon_{x,\text{max}}\big), \tag{S.14}$$
$$\boldsymbol{B}_y := \big(\boldsymbol{D}_y < \epsilon_{y,\text{max}}\big), \tag{S.15}$$
$$\boldsymbol{B}_{xy} = \boldsymbol{B}_x \odot \boldsymbol{B}_y, \tag{S.16}$$

where $\epsilon_{x,\text{min}}$ and $\epsilon_{x,\text{max}}$ are the minimum and maximum possible movement of flowing object in horizontal direction, and $\epsilon_{y,\text{max}}$ is maximum possible object movement in lateral direction (treated as noise).

To find object correspondences that satisfy the Poiseuille flow principles ((S.14)-(S.16)), we defined matching matrices as follows:

$$\boldsymbol{M}_y = \boldsymbol{B}_{xy} \odot (\boldsymbol{D}_y \oplus \boldsymbol{1}), \tag{S.17}$$
$$\boldsymbol{M}_\sigma = \boldsymbol{B}_{xy} \odot \boldsymbol{D}_\sigma, \tag{S.18}$$

where $\odot$ and $\oplus$ refer to element-wise multiplication and addition, respectively. The matching matrix is multiplication of a boolean matrix ($\boldsymbol{B}$) and real (or integer) matrix ($\boldsymbol{D}$). Elements of the matching matrix that do not satisfy the principles are zero, and elements that satisfy the principles are error value as same as $\boldsymbol{D}_y + \boldsymbol{1}$ and $\boldsymbol{D}_\sigma$. Since $y$-directional possition is usually unchaged and $\boldsymbol{D}_y$ is an integer matrix, its error is usually zero. Therefore, integer one is added to each element to prevent that zero error element becomes boolean zero. Final matching matrix is computed as weighted summation of two matching matrices as

$$\boldsymbol{M} = w_1 \boldsymbol{M}_y + w_2 \boldsymbol{M}_\sigma. \tag{S.19}$$

Here, we set $w_1$ as 1 and $w_2$ as 2.

Now, we can find correspondences by searching non-zero elements in $\boldsymbol{M}$. Each column of the matching matrix refers to each object in the current frame and each row of the matching matrix refers to each object in the previous frame; e.g., if the second column of the matching matrix is $[0, 0, 0, 1.2, 0]^T$, then the second object of current frame matches to the fourth object in the previous frame. If the column or row of the matching matrix has two or more non-zero elements, then the smallest value is taken. In the case of Fig. S.5, the correspondences can be represented as follow:

$$\mathcal{C}_{\text{matching}} = \{4^{(n-1)} = 1^{(n)}, 5^{(n-1)} = 3^{(n)}, 6^{(n-1)} = 2^{(n)}, 7^{(n-1)} = 5^{(n)}, 8^{(n-1)} = 6^{(n)}, 9^{(n-1)} = 4^{(n)}\}. \tag{S.20}$$

We also have to consider *in* state of the current frame, and *out* state of the previous frame. If all elements of a column are zeros, then that column refers to *in* state object. If all elements of a row are zeros, then that row refers to *out* state object; in case of Fig. S.5,

$$\mathcal{C}_{\text{out}} = \{1^{(n-1)}, 2^{(n-1)}, 3^{(n-1)}\}, \tag{S.21}$$

$$\mathcal{C}_{\text{in}} = \{7^{(n)}, 8^{(n)}\}. \tag{S.22}$$

We also have to consider occlusion of objects. Height of a micro-channel is usually larger than the size of particles and the microscope just observes horizontal plane; thus, at the top view, flowing particles can vertically overlap in several frames (called occlusion). Therefore, not only *in* and *out* of objects but also *appearance* and *disappearance* should be considered. For example, in the case of Fig. S.6, *in*=F, *out*=A, *appearance*=B (or C), *disappearance*=D (or E), and *matching*=*NULL* at $n$ frame.

To find all states (in, out, appearances and disappearances), we utilized *predicted positions* of each object based on the previous frames and applied it as additional principles as follows:

$$\boldsymbol{B}_{\hat{x}} := (d(\hat{\boldsymbol{p}}_{x,n}, \boldsymbol{p}_{x,n}) < \hat{\epsilon}_{x,max}) \tag{S.23}$$

where

$$\hat{\boldsymbol{p}}_{x,n} = \boldsymbol{p}_{x,n-1} + \hat{\boldsymbol{v}}_{x,n-1}, \tag{S.24}$$

$$\hat{\boldsymbol{v}}_{x,n} = \boldsymbol{p}_{x,n} - \boldsymbol{p}_{x,n-1}, \tag{S.25}$$

$$\hat{\boldsymbol{p}}_{x,n} = 2\boldsymbol{p}_{x,n-1} - \boldsymbol{p}_{x,n-2}. \tag{S.26}$$

Here, $\hat{\epsilon}_{x,\text{max}}$ is maximum error of predicted position in $x$. Using the predicted positions, the matching matrix is updated as follows:

$$\boldsymbol{B}_s := \boldsymbol{B}_{xy} \Leftarrow \boldsymbol{B}_{\hat{x}}, \tag{S.27}$$

$$\boldsymbol{M}_y = \boldsymbol{B}_s \odot (\boldsymbol{M}_y \oplus 1), \tag{S.28}$$

$$\boldsymbol{M}_\sigma = \boldsymbol{B}_s \odot \boldsymbol{M}_\sigma, \tag{S.29}$$

where $\Leftarrow$ refers to a replacement of a block in $\boldsymbol{B}_{xy}$ as $\boldsymbol{B}_{\hat{x}}$.

Let us consider some objects are vanished at $n$ frame that existed at $n-1$ frame. If the predicted $x$-position of the vanished object is larger than the frame width ($W = 500$ in our case), then it may be *out*. If the predicted $x$ position is within the frame, then it may be *disappearance* due to the occlusion.

TABLE S.2

STRUCTURE OF A CELL CLASSIFIER

| No. | Layers | Size (filter, pool) | Stride | Pad | Input size ($H \times W \times D$) |
|---|---|---|---|---|---|
| 1 | Conv - ReLU | 3 | 1 | 0 | $21 \times 21 \times 1$ |
| 2 | Max pooling | 2 | 2 | 0 | $19 \times 19 \times 20$ |
| 3 | Conv - ReLU | 3 | 1 | 0 | $9 \times 9 \times 20$ |
| 4 | Max pooling | 2 | 2 | 0 | $7 \times 7 \times 50$ |
| 5 | Conv - ReLU | 3 | 1 | 0 | $3 \times 3 \times 50$ |
| 6 | FC | - | - | - | $1 \times 1 \times 300$ |
| 7 | Softmax | - | - | - | $1 \times 1 \times 3$ |

* FC: fully connected layers.

We can also consider some objects that appear at $n$ frame that did not exist at $n-1$ frame. If the observed $x$-position is smaller than $\epsilon_{x,\text{max}}$, then it may be *in*. On the other hand, if the observed $x$-position is larger than $\epsilon_{x,\text{max}}$, then it may be *appearance*. All these states are formulated as follows:

$$\textit{out:}\ W - \hat{\mu}_{x,n} \leq \epsilon_{x,\text{max}} \text{ when } \mu_{x,n-1} \text{ is vanished}$$
$$\textit{disapp.:}\ W - \hat{\mu}_{x,n} > \epsilon_{x,\text{max}} \text{ when } \mu_{x,n-1} \text{ is vanished}$$
$$\textit{in:}\ \mu_{x,n} \leq \epsilon_{x,\text{max}} \text{ when } \mu_{x,n} \text{ is new one}$$
$$\textit{app.:}\ \mu_{x,n} > \epsilon_{x,\text{max}} \text{ when } \mu_{x,n} \text{ is new one}$$

By considering all these principles, tracking algorithm enables robust tracking of multiple objects including occlusions. Even though there is detection error on the predicted density map and flattening algorithm, the tracking algorithm can compensate wrong object counts during the tracking.

## S.4. Detection and Localization Accuracy

The flattening algorithm can perform both detection and localization tasks. The integration error in Fig. S.2(b) represents detection accuracy of CellNet. We evaluated localization accuracy by the extraction accuracy of flattening. Totally 560 beads in 100 image frames were used to evaluate the localization accuracy. The position errors are $e_{pos,x} = 0.35 \pm 0.24$ pixels and $e_{pos,y} = 0.41 \pm 1.37$ pixels, respectively. Lastly, the estimated radius ($3\sigma$) error is $e_{pos,3\sigma} = 0.66 \pm 0.85$ pixels.

## References


[1] W. Xie, J. A. Noble, and A. Zisserman, "Microscopy cell counting and detection with fully convolutional regression networks," *Computer Methods in Biomechanics and Biomedical Engineering: Imaging & Visualization*, pp. 1–10, 2016.

[2] J. Long, E. Shelhamer, and T. Darrell, "Fully convolutional networks for semantic segmentation," in *Proceedings of the IEEE Conference on Computer Vision and Pattern Recognition*, 2015, pp. 3431–3440.

[3] A. Vedaldi and K. Lenc, "Matconvnet: Convolutional neural networks for matlab," in *Proceedings of the 23rd ACM international conference on Multimedia*. ACM, 2015, pp. 689–692.
